# Transpancreatic precut sphincterotomy with a novel highly rotatable sphincterotome in balloon enteroscopy-assisted endoscopic retrograde cholangiopancreatography

**DOI:** 10.1055/a-2584-1703

**Published:** 2025-05-06

**Authors:** Tadahisa Inoue, Rena Kitano, Tomoya Kitada, Kazumasa Sakamoto, Satoshi Kimoto, Jun Arai, Kiyoaki Ito

**Affiliations:** 1 12703 Department of Gastroenterology, Aichi Medical University, Nagakute, Japan

Precut techniques are commonly employed as salvage methods in cases of difficult biliary cannulation during endoscopic retrograde cholangiopancreatography (ERCP). When pancreatic duct cannulation is successful, transpancreatic precut sphincterotomy (TPS) is considered a reasonable option 1 . However, during balloon enteroscopy-assisted ERCP (BE-ERCP) for surgically altered anatomy, obtaining a clear view of the duodenal papilla is often challenging. Additionally, using a forward-viewing scope, the absence of a forceps elevator, and the approach from the anal side complicate the orientation of the sphincterotome blade in the correct cutting direction 2 .

Recently, a sphincterotome with enhanced rotational capability and wide blade mobility has become available 3 ( Fig. 1 ). This feature may facilitate adjustment of the blade’s direction, even in BE-ERCP cases, enabling effective TPS in difficult biliary cannulation scenarios.

**Fig. 1:**
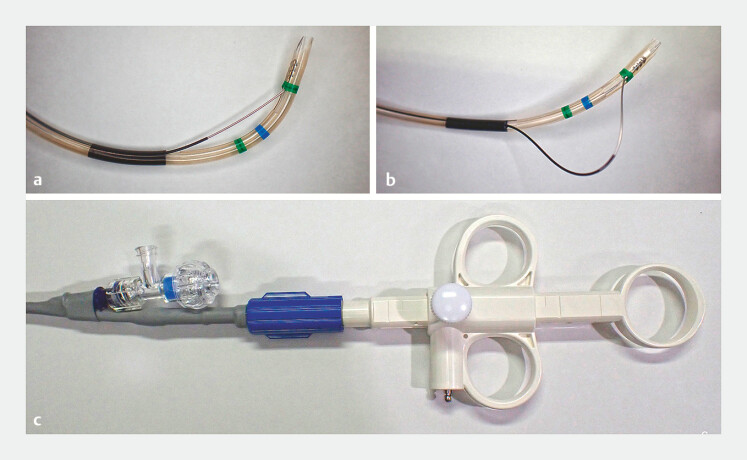
**a, b** The novel sphincterotome (ENGETSU; KANEKA Medix) enables precise blade movement through push-and-pull operations on the handle. **c** The handle is also rotatable, allowing 360-degree control over the blade’s orientation.

Here, we report two cases, a 69-year-old man and a 72-year-old woman, in whom this novel sphincterotome was successfully used for TPS during BE-ERCP. Both patients had undergone Roux-en-Y gastrectomy and presented with obstructive jaundice caused by common bile duct (CBD) stones. A short-type single-balloon enteroscope was advanced to the duodenal papilla in both cases, but biliary cannulation was unsuccessful. Pancreatic duct cannulation was achieved, allowing transition to the double-guidewire technique, although biliary cannulation remained unsuccessful.

Therefore, TPS was attempted using the novel rotatable sphincterotome. In both cases, the blade was initially oriented incorrectly, but after rotational adjustment, the blade aligned with the bile duct axis, enabling effective and precise incision ( Fig. 2 and Fig. 3 , Video 1 ). Following the incision, biliary cannulation was successfully achieved using the double-guidewire technique, and CBD stones were completely removed. Neither patient experienced adverse events, and their symptoms resolved rapidly.

**Fig. 2:**
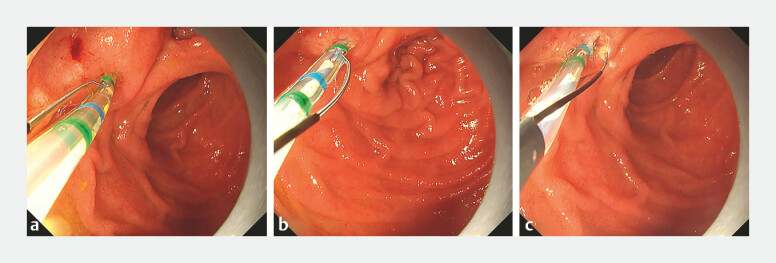
**a** In Case 1, upon initial insertion of the sphincterotome, the blade was oriented opposite to the bile duct axis and the oral protrusion of the papilla. **b** Using the sphincterotome’s rotational capability, the blade was realigned to the correct direction. **c** This adjustment enabled a precise transpancreatic precut sphincterotomy, leading to successful biliary cannulation using the double-guidewire technique.

**Fig. 3:**
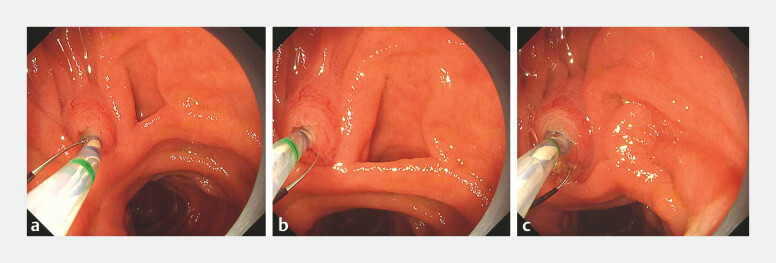
**a** In Case 2, upon initial insertion of the sphincterotome, the blade was misaligned with both the bile duct axis and the oral protrusion of the papilla. **b** After rotating the sphincterotome, the blade was adjusted to the correct orientation. **c** This adjustment enabled a precise transpancreatic precut sphincterotomy, resulting in successful biliary cannulation using the double-guidewire technique.

Transpancreatic precut sphincterotomy using a highly rotatable sphincterotome during balloon enteroscopy-assisted ERCP in patients with Roux-en-Y gastrectomy.Video 1

TPS with this novel rotatable sphincterotome may serve as a valuable salvage technique for difficult biliary cannulation during BE-ERCP. Further studies are warranted to validate its efficacy and safety in larger patient cohorts.

Endoscopy_UCTN_Code_TTT_1AR_2AC

## Notes

Endoscopy E-Videos https://eref.thieme.de/e-videos
*E-Videos* is an open access online section of the journal *Endoscopy* , reporting on interesting cases and new techniques in gastroenterological endoscopy. All papers include a high-quality video and are published with a Creative Commons CC-BY license. Endoscopy E-Videos qualify for HINARI discounts and waivers and eligibility is automatically checked during the submission process. We grant 100% waivers to articles whose corresponding authors are based in Group A countries and 50% waivers to those who are based in Group B countries as classified by Research4Life (see: https://www.research4life.org/access/eligibility/ ). This section has its own submission website at https://mc.manuscriptcentral.com/e-videos .
